# T-cell responses to human papillomavirus type 16 among women with different grades of cervical neoplasia

**DOI:** 10.1038/sj.bjc.6602679

**Published:** 2005-06-28

**Authors:** J C Steele, C H Mann, S Rookes, T Rollason, D Murphy, M G Freeth, P H Gallimore, S Roberts

**Affiliations:** 1 Cancer Research UK Institute for Cancer Studies, University of Birmingham, Edgbaston, Birmingham B15 2TT, UK; 2 Department of Gynaecological Oncology, Birmingham Women's Hospital, Edgbaston, Birmingham B15 2TG, UK; 3 Department of Pathology, Birmingham Women's Hospital, Edgbaston, Birmingham B15 2TG, UK; 4 Department of Gynaecological Oncology, New Cross Hospital, Wolverhampton, West Midlands WV10 0QP, UK; 5 Department of Histopathology, New Cross Hospital, Wolverhampton, West Midlands WV10 0QP, UK

**Keywords:** T cells, CIN, disease grade, HPV16, ELISPOT

## Abstract

Infection with high-risk genital human papillomavirus (HPV) types is a major risk factor for the development of cervical intraepithelial neoplasia (CIN) and invasive cervical carcinoma. The design of effective immunotherapies requires a greater understanding of how HPV-specific T-cell responses are involved in disease clearance and/or progression. Here, we have investigated T-cell responses to five HPV16 proteins (E6, E7, E4, L1 and L2) in women with CIN or cervical carcinoma directly *ex vivo*. T-cell responses were observed in the majority (78%) of samples. The frequency of CD4+ responders was far lower among those with progressive disease, indicating that the CD4+ T-cell response might be important in HPV clearance. CD8+ reactivity to E6 peptides was dominant across all disease grades, inferring that E6-specific CD8+ T cells are not vitally involved in disease clearance. T-cell responses were demonstrated in the majority (80%) of cervical cancer patients, but are obviously ineffective. Our study reveals significant differences in HPV16 immunity during progressive CIN. We conclude that the HPV-specific CD4+ T-cell response should be an important consideration in immunotherapy design, which should aim to target preinvasive disease.

Cervical carcinoma is the second most common female cancer worldwide, with 400 000 new cases diagnosed each year (Boyle, 1997; Landis *et al*, 1998). Infection by human papillomavirus (HPV) is a major risk factor for the development of invasive cervical carcinoma and its precursor cervical intraepithelial neoplasia (CIN). Nearly all (99.7%) cervical cancers are positive for HPV DNA, with HPV type 16 (HPV16) being the most prevalent and present in up to 70% of cervical cancers regardless of geographical origin (Bosch *et al*, 1995; Walboomers *et al*, 1999).

It is clear that there are effective host defence mechanisms against HPVs. Genital infection with HPV is generally transient, with the majority of individuals showing clearance of the virus within 1 year of detection (Evander *et al*, 1995; Ho *et al*, 1998; Woodman *et al*, 2001; Sellors *et al*, 2003). In fact, only a minority of women develop persistent infections with focally high levels of HPV DNA, and only some of these progress to high-grade disease and invasive carcinoma (Herrero *et al*, 2000). Cell-mediated immunity is believed to be critical in the resolution and control of HPV. HIV-infected patients show multiple recurrences of cervical HPV infections (Fruchter *et al*, 1998) and an increased incidence of genital warts (Fennema *et al*, 1995) that appear to reflect an increased risk of progression from subclinical to clinical infection (Chirgwin *et al*, 1995).

There are several studies providing information about CD4+ T-cell responses to HPV16. Using either fusion proteins, panels of overlapping peptides, or virus-like particles (VLPs), CD4 responses to HPV16 E6 (Cubie *et al*, 1989; Strang *et al*, 1990; Nakagawa *et al*, 1996; Tsukui *et al*, 1996; Kadish *et al*, 1997, 2002; Welters *et al*, 2003; de Jong *et al*, 2004), E7 (Cubie *et al*, 1989; Strang *et al*, 1990; Altmann *et al*, 1992; Kadish *et al*, 1994, 1997, 2002; de Gruijl *et al*, 1996a, 1996b, 1998; Luxton *et al*, 1996; Nakagawa *et al*, 1996; Tsukui *et al*, 1996; Hopfl *et al*, 2000; van der Burg *et al*, 2001; Welters *et al*, 2003), E2 (Bontkes *et al*, 1999; de Jong *et al*, 2002, 2004; Welters *et al*, 2003), E5 (Gill *et al*, 1998), E4 (Cubie *et al*, 1989; Nakagawa *et al*, 1996) and L1 (Strang *et al*, 1990; Nakagawa *et al*, 1996; Shepherd *et al*, 1996; Luxton *et al*, 1997; de Gruijl *et al*, 1999) have been demonstrated in both patient and healthy control populations. No clear pattern has yet emerged as to which of these responses, if any, might be associated with regression or progression of disease as only a limited number of prospective studies have been carried out.

The role of naturally occurring cytotoxic T cells (CTLs) in mediating regression of HPV-related disease has not been proven. Using a variety of restimulation protocols, HPV16 E2-, E6- and E7-specific CTLs can be detected in patients with previous (Nakagawa *et al*, 1997) or ongoing HPV infections (Alexander *et al*, 1996; Borysiewicz *et al*, 1996; Evans *et al*, 1996, 1997; Ressing *et al*, 1996; Jochmus *et al*, 1997; Konya *et al*, 1997; Nimako *et al*, 1997). Most of this work has involved the use of selected peptides bound to the surface of various target cells to restimulate CD8 responses *in vitro*. As such, these experiments are restricted to only a few HLA alleles using peptides that may not be representative of HPV epitopes expressed during natural infection. Studies using whole HPV proteins to restimulate CTL responses, either in a soluble form (Nakagawa *et al*, 1997) or expressed by recombinant viral vectors (Nimako *et al*, 1997), where the whole protein is naturally processed, have been more successful. Autologous dendritic cells (DCs) presenting HPV16 peptides or whole proteins have also been used to restimulate E6- and E7-specific CTL responses from both normal donors and women with cervical carcinoma *in vitro* (Murakami *et al*, 1999; Schoell *et al*, 1999; Thornberg *et al*, 2000; Davidson *et al*, 2001; Liu *et al*, 2001; Chiriva-Internati *et al*, 2002; Valdespino *et al*, 2005), although it is not clear whether these represent primary or memory T-cell responses. Overall, the results suggest that naturally occurring HPV E6- and E7-specific CTLs do exist in patients with HPV-associated disease, although their detection may depend on the methods used to reactivate them *in vitro*. More recently, using tetramer technology, it has been shown directly that HPV16 E7-specific CTLs do exist in the peripheral blood of women with high-grade CIN and cervical carcinoma, but they are extremely rare (Youde *et al*, 2000). Very little is known about the HPV16-specific CTL response to proteins other than E6 and E7.

The link between HPV16 and cervical carcinoma opens up the possibility of immune T-cell intervention, either against the preinvasive lesions from which tumours arise, or against the virus antigen-positive tumour cells themselves. To effect such strategies will require a better understanding of the spectrum of T-cell responses induced by HPV16 antigens during the course of natural infection, and of their role in disease clearance and/or progression. Much of the work to date has understandably focused on responses to the E6 and E7 transforming proteins, since these are the only viral antigens constitutively expressed in HPV-positive tumour cells. By contrast, there has been no concerted effort to look at the totality of responses to a single agent, including responses to viral antigens that are expressed later in the replication cycle that may be useful targets for the treatment of preinvasive lesions. In this study, we have used CD4- and CD8-depleted populations of responder cells in an ELISPOT assay of IFN-*γ* release to screen directly *ex vivo* for both CD4+ and CD8+ T-cell responses to five HPV16 proteins (E6, E7, E4, L1 and L2) from a cohort of 41 women with varying grades of cervical disease. The frequency, magnitude and antigen specificity of the responses obtained are discussed in relation to disease severity.

## MATERIALS AND METHODS

### Patients and controls

A total of 41 women attending colposcopy clinics or undergoing surgery for cervical disease (Department of Gynaecological Oncology, Birmingham Women's Hospital, Birmingham, UK) were investigated. Ethical approval for this study was obtained from the South Birmingham Research Ethics Committee (Study Number 5147) and informed consent was obtained from all subjects. Abnormal cervical cytology was diagnosed and confirmed by histopathology. Patients were assigned to one of four groups according to the histology reports obtained from cervical tissue taken at the time they were bled: those reported to have no evidence of CIN, those with low-grade disease (CIN I), high-grade disease (CIN II/III) and those with cervical carcinoma. The clinical details are shown in Table 1.

The HPV genotype of patient biopsies obtained during the course of this study could not be established. However, the transient nature of genital HPV infections questions the validity of HPV typing at a single time point, since this may not necessarily reflect a past history of HPV infection in a significant proportion of women, especially those with low-grade disease (Woodman *et al*, 2001). High-grade disease would be expected to be associated with a more persistent HPV infection.

Nine age-matched female virgins were recruited as negative controls. Virginity was established by gynaecologists who obtained full clinical and sexual histories. Normal healthy donors were considered not suitable as a negative control group, since the evidence suggests that a significant number will have encountered this virus (Woodman *et al*, 2001). Indeed, many recent studies have demonstrated HPV16-specific T-cell responses in normal donors (de Jong *et al*, 2002, 2004; Welters *et al*, 2003) and HPV16 is common in women with normal cytology (Bosch *et al*, 1995). Therefore, virgins are the control group most likely to represent HPV16 negativity, although it is not possible to establish whether this is true in every case.

### Preparation of CD4-enriched and -enriched T-cell populations

Unfractionated mononuclear (UM) cells were separated from 40–60 ml heparinised blood samples by isopycnic centrifugation on lymphocyte separation medium (Lymphoprep; Nycomed, Oslo, Norway) and cryopreserved in liquid nitrogen. CD4+ and CD8+ T-cell depletions were carried out on UM cells using antibody-coated magnetic beads (Dynal, Oslo, Norway) according to the method previously described (Steele *et al*, 2002). Depleted populations were used directly as responder cells in the ELISPOT assay. FACS analysis using dual-labelled anti-human CD8:FITC/anti-CD4:RPE, or the isotype-matched negative control IgG1:FITC/IgG1:RPE (Serotec, UK) was carried out each time to verify the depletions, and in order to calculate the number of T cells of the relevant phenotype being added to each well.

### Preparation of HPV16 VLPs for use in the ELISPOT assays

A recombinant baculovirus containing the HPV16 L1 and L2 genes was kindly provided by Professor M Stanley (Department of Pathology, University of Cambridge). HPV16 VLPs were produced following the infection of SF9 insect cells, and were purified on caesium chloride gradients according to published methods (Cubie *et al*, 1998).

### ELISPOT assay of IFN-*γ* release

Overlapping synthetic peptides (30–35mers; Alta Bioscience, Birmingham, UK) covering the entire primary sequences of the HPV16 E6, E7 and E4 proteins (overlapping by 14–16 amino acids; sequences shown in Table 2), and baculovirus-expressed HPV16 VLPs comprising both L1 and L2 were used to screen for CD4+ and CD8+ T-cell responses using an ELISPOT assay of IFN-*γ* release (ELISPOT assay for human interferon-*γ*; Mabtech, Sweden). The ELISPOT assay procedure has been published previously (Steele *et al*, 2002). Briefly, CD4- or CD8-enriched responder cell populations were used at 4 × 10^5^ cells per well (in duplicate) and peptides were added to a final concentration of 10 *μ*g ml^−1^. A negative control well with no peptide or VLP and a positive control well containing cells and 0.2% PHA-P (Difco Laboratories) instead of peptide were included for every sample. The plates were counted using an automated system (AID, Strasbourg, Germany) and background counts obtained in the absence of peptide or VLP were subtracted. An ELISPOT response among the patient group was only considered positive if the number of spots obtained fell above a negative cutoff value. These were calculated for every peptide and the VLP preparation, and taken as 2 standard deviations (s.d.) above the mean of the counts obtained using the negative control group comprising nine age-matched female virgins.

### Enzyme-linked immunosorbent assay (ELISA) to determine HPV16 antibody levels

Purified baculovirus-expressed HPV16 VLPs (a kind gift from Professor Martin Sapp, University of Mainz, Mainz, Germany) were used in an ELISA to determine HPV16-specific antibody levels in plasma samples obtained from each subject. This source of VLPs was unsuitable for use in the ELISPOT assay due to problems with toxicity. An ELISA method for detection of HPV1 VLP-specific antibodies has been published by us recently (Steele *et al*, 2002). The procedure used here for HPV16 VLPs was the same, except that a biotinylated second antibody and a peroxidase-labelled StreptABComplex (Dako) were employed to increase the sensitivity. Antibody levels were quantitated by relating the absorbancies to that obtained from the positive control, an anti-HPV16 L1 antibody, (Camvir 1, Abcam, UK) run under standard conditions on every assay plate. The negative cutoff value was taken as two standard deviations above the mean of the absorbencies obtained using the virgin controls.

## RESULTS

To define the frequency and spectrum of T-cell responses to HPV16, we used five HPV16 proteins as targets; E6 and E7 are the main transforming proteins and are expressed early in the infection cycle, E4 is an abundant protein that is expressed at an intermediate and late stage, and the capsid proteins L1 and L2 appear at later stages of the life cycle. Overlapping synthetic peptides covering the entire primary sequence of E6, E7 and E4 (30–35mers; sequences are shown in Table 2) were used in an ELISPOT assay of IFN-*γ* release. It is important to note that similar-length peptides have been used successfully in other human immunological studies, including ELISPOT assays, to detect HPV-specific T-cell responses (van der Burg *et al*, 2001; de Jong *et al*, 2002, 2004; Welters *et al*, 2003). Human papillomavirus 16 VLPs were used as a source of L1 and L2 antigens in the ELISPOT assay.

### T-cell reactivity was detected in the majority of patients

**Figure 1:**
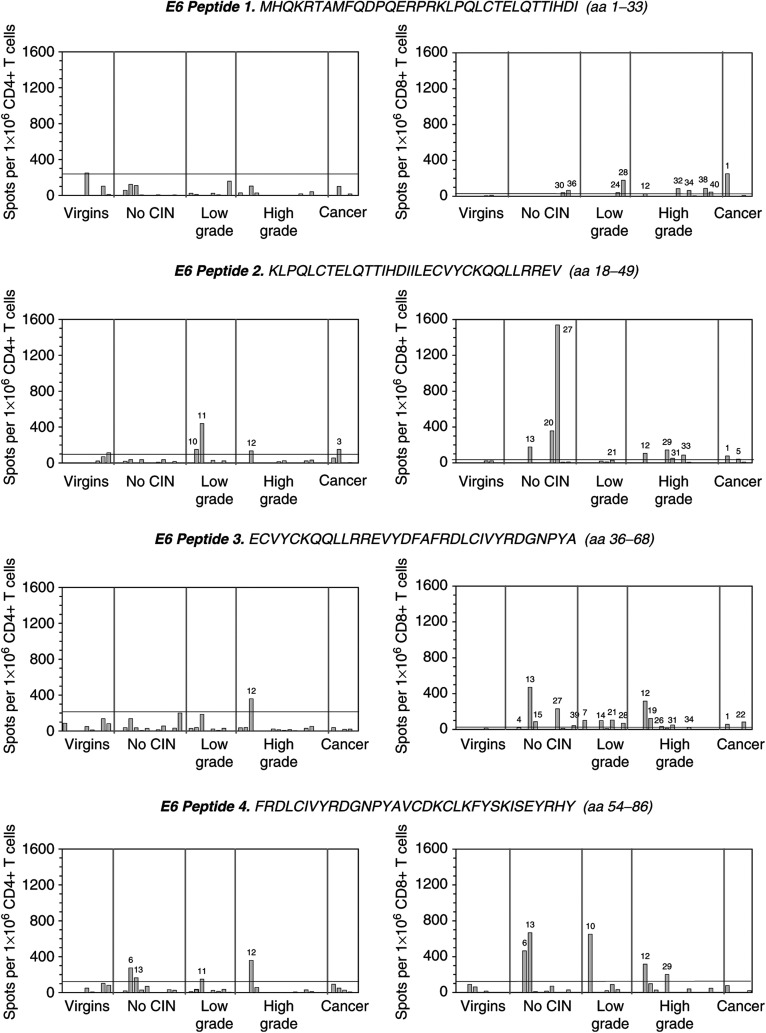

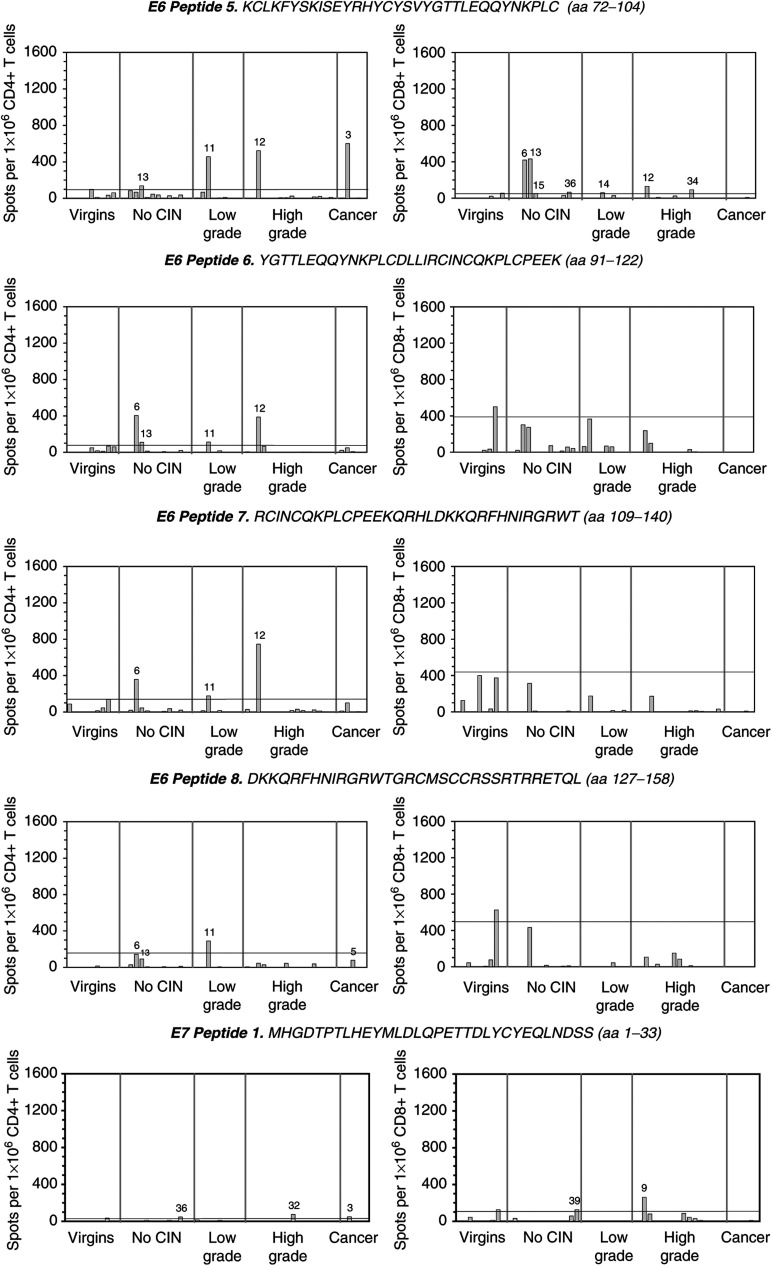

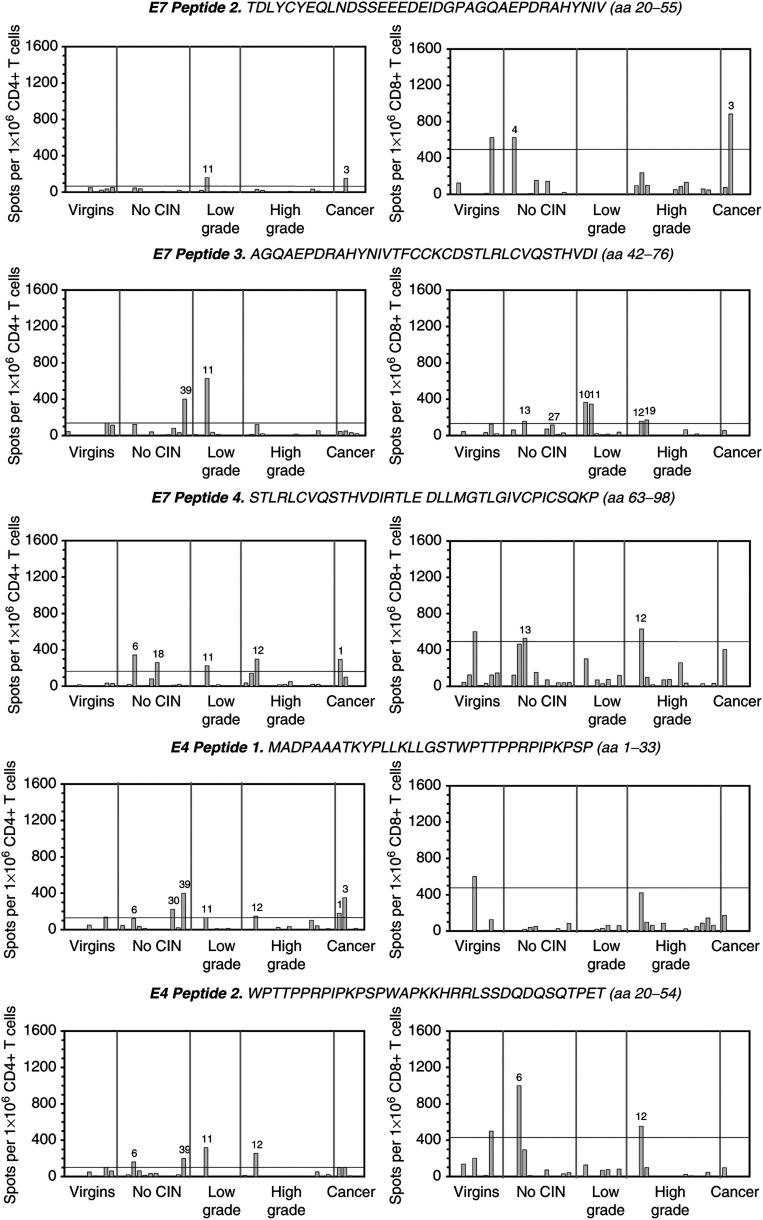

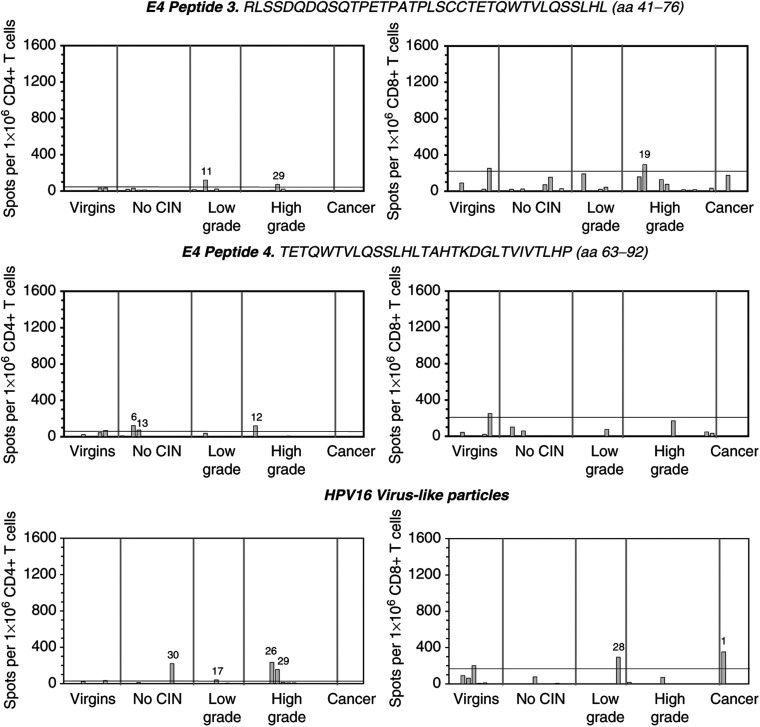
CD4+ (on the left side of the figure) and CD8+ (on the right side of the figure) T-cell reactivities to HPV16 E6, E7, E4 peptides and HPV16 virus-like particles. CD4+ and CD8+ enriched responder cell populations from each patient were screened against 30–35mer peptides (overlapping by 14–16 amino acids) covering the entire primary sequences of the HPV16 E6, E7 and E4 proteins (sequences shown using an ELISPOT assay of interferon-*γ* release). The assay employed 4 × 10^5^ responder cells well^−1^ (in duplicate), and both the peptides and the VLPs were used at a concentration of 10 *μ*g ml^−1^. Responses obtained from the negative control group comprising nine age-matched female virgins were used to establish the negative cutoff values for each peptide and the VLPs (mean+2 s.d.). Positive responses are those which fall above the negative cutoff value (—), and the patient number is indicated. Results are expressed as the number of spots obtained per 1 × 10^6^ T cells of the appropriate phenotype added to each well. These figures were calculated following FACS analysis of depleted cell samples. Any background reactivity in the absence of peptide or VLPs has been subtracted.

**Figure 2:**
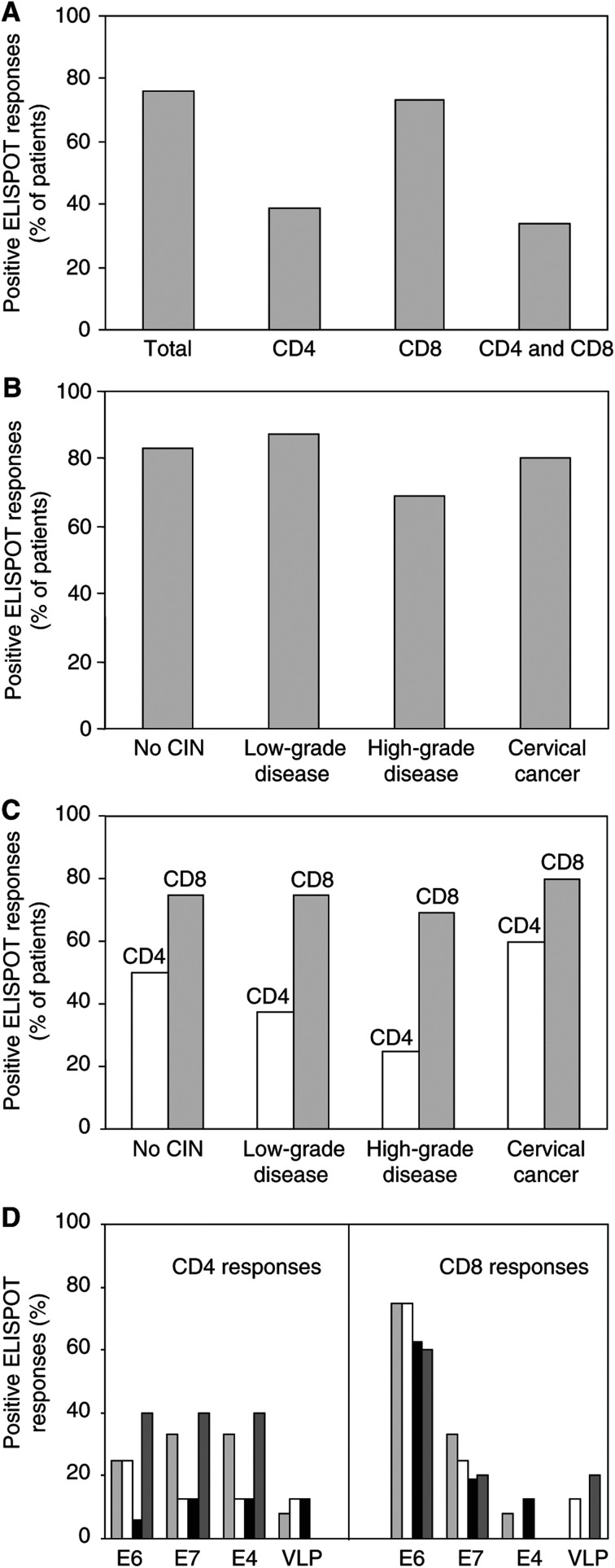
Summary of the ELISPOT assay data. The percentages of positive ELISPOT responses obtained generally (**A**), in relation to disease grade (**B**), CD4 and CD8 responses in relation to disease grade (**C**), and the antigen specificities of the CD4+ and CD8+ T-cell reactivities in relation to disease grade (**D**). ▒ represents no CIN, □ low-grade disease, ▓ high-grade disease, and ▪ cervical cancer.

To determine whether responding IFN-*γ*-secreting T cells in the ELISPOT were CD8+ or CD4+, peripheral blood lymphocytes were negatively depleted using magnetic beads. FACS analysis of the CD4- or CD8-depleted T-cell populations revealed that in nearly all cases there was a depletion of greater than 99% of the appropriate T-cell subset (data not shown). The results of the ELISPOT assays are shown in Figure 1 and the data are summarised in Figure 2. We demonstrated either CD4+ or CD8+ T-cell reactivity in 78% (32 out of 41) of the patient samples tested, with 34% (14 out of 41) showing both CD4 and CD8 responses (Figure 2A). Among the various disease grades, the frequency of ELISPOT responders overall was: no CIN, 83%; low-grade disease, 87.5%; high-grade disease, 69%; cervical cancer, 80% (Figure 2B).

The majority of the control samples showed little or no T-cell reactivity, with the exception of numbers 5 and 9, both of which demonstrated responses to a few peptides, particularly those covering HPV16 E6 (Figure 1). Although this increased the negative cutoff point quite considerably in some cases, it would be incorrect to eliminate these samples from the study.

### CD8 responses were observed more often than CD4 responses

The results of the ELISPOT assays showed that CD8+ T-cell reactivity occurred at almost twice the frequency of CD4+ reactivity (Figure 2A). CD4 responses were detected in 39% (16 out of 41) of patients, and CD8 responses in 73% (30 out of 41). A similar percentage of women gave CD8+ T-cell responses in each patient group: no CIN (75%; nine out of 12), low-grade disease (75%; six out of eight), high-grade disease (69%; 11 out of 16) and cervical cancer (80%; four out of five) (Figure 2C). In contrast, CD4+ responses were more frequent among the women with no CIN (50%; six out of 12) or with cervical cancer (60%; three out of five), than among those with low-grade (37.5%; three out of eight) and high-grade disease (25%; four out of 16) (Figure 2C). It is the decreased number of responders in these last two groups, particularly those with high-grade disease, which is responsible for our finding that CD4+ T-cell reactivity was much less common overall.

There was no significant difference in the magnitude of CD4 or CD8 responses obtained among patient groups (Figure 1). The greatest CD4+ T-cell response (746 spots per 1 × 10^6^ CD4+ T cells) was obtained from a woman with high-grade disease (patient 12), using an E6 peptide (RCINCQKPLCPEEKQRHLDKKQRFHNIRGRWT; aa 109–140). In a CD8 response, the highest frequency of spots (1538 spots per 1 × 10^6^ CD8+ T cells) was obtained from a patient with no CIN (patient 27), again in response to an E6 peptide (KLPQLCTELQTTIHDIILECVYCKQQLLRREV; aa 18–49).

### CD8 T-cell responses to HPV16 E6 are dominant

Differences in the antigen specificities of the CD4 and CD8 responses were also observed in the ELISPOT assays, although this was not related to disease grade (Figure 2D). A similar number of responses to E6 (50%), E7 (56%) and E4 (56%) peptides was seen among the CD4-positive samples, but only 25% responded to VLPs. There was, however, a very dominant CD8+ T-cell response to peptides covering E6, with 93% of the CD8-positive samples showing reactivity to this protein. CD8 responses to the other proteins were far less common (E7 33%; E4 10%; VLP 7%). For both CD4+ and CD8+ T-cell reactivity, many of the patients responded to more than one protein and saw more than one peptide on each (Figure 1). This was particularly noticeable with patients 6 and 13 (no CIN), 11 (low-grade disease) and 12 (high-grade disease).

### Seropositivity increases with disease severity and is related to positive T-cell reactivity

**Figure 3:**
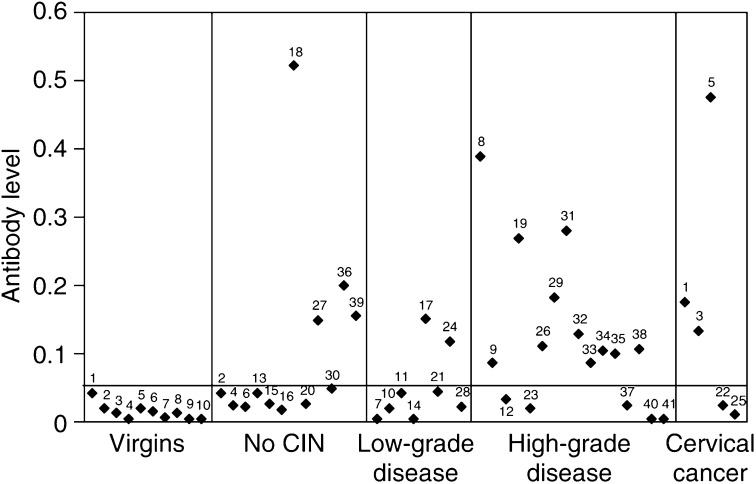
Relative HPV16-specific antibody levels obtained using an ELISA that employed HPV16 L1-VLPs. Antibody levels were quantitated by relating the absorbencies obtained for each sample to that obtained from the positive control (Camvir 1) run under standard conditions on every assay plate. The negative control providing the negative cutoff value (—) was taken as 2 s.d. above the mean of the absorbencies obtained using the virgin controls. Background readings, where no antigen had been bound to the plate, were subtracted from experimental readings in every case.

The serological status of all subjects in the study was assessed in order to obtain additional information about HPV16 immune status. Antibodies to HPV16 VLPs were detected in 20 out of the 41 (48.7%) plasma samples overall (Figure 3). In women with no CIN and low-grade disease, only 33 and 25% had positive serology, respectively. In contrast, higher frequencies of seropositivity were seen among the women with high-grade disease (69%) and cervical cancer (60%). The analysis revealed an increase in the frequency of antibody positivity (but not titre) with disease severity. The median antibody levels obtained were 0.04 (range: 0.025–0.52) for the women with no CIN, 0.03 (range: 0–0.145) for those with low-grade disease, 0.0975 (range: 0–0.385) for those with high-grade disease and 0.12 (range: 0.015–0.48) for the cervical cancer patients. The differences in median values among the disease groups were not statistically significant (*P*-values all greater than 0.187; Mann–Whitney test). All of the patient groups had significantly higher titres than those obtained from the negative control group of female virgins (*P*-values all less than 0.017; Mann–Whitney test), with the exception of those women with low-grade disease (*P*=0.091).

Out of the 20 seropositives obtained in this study, 18 (90%) showed either CD4+ or CD8+ T-cell reactivity to HPV16 peptides in the ELISPOT assays. In all, 15 samples (71%) out of the 21 with negative serology also gave a T-cell response. These samples were from patients who fell predominantly into the no CIN or low-grade disease groups. Interestingly, the patients who demonstrated T-cell responses to multiple peptides and proteins (patients 6, 11, 12 and 13) (Figure 1) were seronegative, whereas the samples with the highest antibody titres (patients 5, 8, 18, 18 and 31) were either negative in the ELISPOT assay, or demonstrated T-cell responses to only a narrow spectrum of peptides.

## DISCUSSION

Using CD4- and CD8-enriched populations of responder cells isolated from 41 women with varying degrees of cervical disease (Table 1), we have investigated the frequency and spectrum of HPV16-specific CD4+ and CD8+ T-cell responses, using ELISPOT assays of IFN-*γ* release, to HPV16 E4, E6, E7, L1 and L2. Significantly, in this study, cells were used directly *ex vivo* in the ELISPOT assay, without undergoing any kind of *in vitro* restimulation. We demonstrated either CD4+ or CD8+ T-cell reactivity in the majority of the patient samples tested (78%), with 34% showing both CD4 and CD8 responses. There was little difference in the magnitude of the responses obtained between the CD4- or CD8-enriched populations (Figure 1), and among the various disease grades we found no significant difference in the frequency of ELISPOT responders overall (Figure 2B). The high detection rate of responses *ex vivo* was encouraging, considering the numerous reports suggesting that HPV-specific T cells are rare in peripheral blood. This is probably due to the high sensitivity of the ELISPOT assay, and the fact that the study was not restricted to selected peptides with specific HLA restrictions, that may lower the rate of detection.

### Human papillomavirus-specific CD4+ T cells may play a critical role in disease clearance

An effective CTL response might be important for HPV clearance. Human papillomavirus 16-specific CTLs are more frequent in women with cleared infection than those with newly diagnosed Human papillomavirus 16-positive CIN (Nakagawa *et al*, 1997). However, both CD4 and CD8 effectors have been shown to be involved (Nakagawa *et al*, 2002). Our observation that the frequency of CD4 responders varies among the disease grades suggests that the CD4+ T-cell response might be critically involved in HPV clearance. The patients with low- and high-grade disease showed CD4 reactivity less frequently. These women are more likely to have progressive disease than those patients with no CIN, and it is possible that their lack of CD4 T-cell reactivity is contributing to progression. Notably, another study has used similar protocols to examine T-helper responses to HPV16 E2, E6 and E7 peptides in healthy individuals and in women with cervical carcinoma (Welters *et al*, 2003). CD4 reactivity was found to be far more common among the group of normal donors than the cancer patients, again suggesting that HPV16-specific CD4+ T-cell immunity is important for disease protection. Evidence from allograft recipients and HIV-infected individuals (Palefsky *et al*, 1999) also indicates that it is the absolute deficit in CD4+ T cells which is the important risk factor for HPV-induced disease and associated neoplastic progression in the immunocompromised individual, and CD4+ T cells have been shown to be prominent in resolving cutaneous (Iwatsuki *et al*, 1986) and genital warts (Coleman *et al*, 1994). Other recent studies looking for HPV-specific T cells in peripheral blood also suggest that helper T cells play a central role in the control of HPV infection, disease regression and clearance (Kadish *et al*, 2002; de Jong *et al*, 2004).

### Human papillomavirus-specific immunity is different in cancer

We found the highest number of CD4 responders in the small group of patients with cancer. This is in agreement with some reports (de Gruijl *et al*, 1996a, 1996b, 1998; de Jong *et al*, 2004), but not others, where CD4 responses have been found to be decreased in cancer (Luxton *et al*, 1996, 1997; Nakagawa *et al*, 1996; Hopfl *et al*, 2000; van der Burg *et al*, 2001; Welters *et al*, 2003). However, all of these studies use different methodologies and the majority used *in vitro* restimulation protocols. There seems to be little doubt that T-cell responses in patients clearing cervical HPV infection are different from those progressing to cervical cancer. It is conceivable that an ineffective HPV-specific CD4+ T-cell response early during infection will allow HPV to persist and the establishment of high-grade disease. However, it seems that the presence of a tumour will eventually induce CD4+ T-cell immunity. This could be because the tumour will eventually breach the basement membrane of the epithelium and viral antigens will become exposed to the immune system. In invasive carcinoma, there will also be an increase in the amount of infected tissue and subsequently viral load. Indeed, HPV16 responses have been shown to be dependent on antigen dose in experiments using a murine model in which viral antigen is expressed in keratinocytes and mimics the natural route of infection (Chambers *et al*, 1994). A surprisingly high frequency of HPV-specific CD4+ T-cell responses in women with cervical carcinoma compared to those with high-grade CIN has also been observed in a similar study published recently by de Jong *et al* (2004). They also looked at cytokine production and their results suggested that cervical cancers do not provide the appropriate proinflammatory environment for the induction of a potent and well-polarised T-cell response, and that if CD4+ T-cell priming occurs at this stage of disease it will most likely result in an ineffective antitumour response.

### CD8+ T-cell responses to HPV16 E6 are dominant

Differences in the antigen specificities of the CD4 and CD8 responses were also observed in this study. There was a very dominant CD8+ T-cell response to peptides covering HPV16 E6 (Figure 1A), a protein known to be critical for malignant transformation and maintenance of the transformed phenotype. It could be concluded from our results that E6-specific CD8+ T cells do not play a major role in HPV clearance because they were so predominant across all disease grades (Figure 2C). This is supported by other studies where HPV16 E6-specific CTLs have been shown to be associated with disease and viral persistence (Bontkes *et al*, 2000; Nakagawa *et al*, 2000).

### Antigen specificity and disease severity are unrelated

There was no obvious relationship between antigen specificity and disease severity for either CD4+ or CD8+ T-cell reactivities. This may be explained by the fact that the immune system is probably primed early on during productive infection when all of the HPV16 proteins are being expressed. Human papillomavirus 16 infection of the cervix is believed to become less productive as the disease progresses, and, following malignant transformation when HPV DNA becomes integrated in the host genome (Cullen *et al*, 1991), the expression of all viral proteins, other than E6 and E7, ceases. However, HPV16 proteins are still being expressed in high-grade lesions (Doorbar *et al*, 1997) and it is likely that HPV-specific memory T cells are already present.

### Cross-reacting T-cell responses

Given the degree of sequence homology between related HPV types, it is possible that we may have detected crossreactive responses to other genital HPVs. T-cell crossreactivity usually requires conserved blocks of sequence identity and this would be expected to restrict the number of epitopes which would be shared between virus types. Crossreaction is most likely to be a problem with L1, which is the most highly conserved of the HPV proteins tested. However, we have employed VLPs that contain type-specific epitopes to screen for L1 and L2 responses. E6 and E7 proteins only exhibit approximately 40% sequence homology between related virus types, and E4 sequences are highly type-specific. The problem of crossreaction has been thoroughly investigated in our recent study using HPV1 (Steele *et al*, 2002), and we believe that the responses obtained here are HPV16-specific. If the problem of crossreaction with the common cutaneous virus types existed, this would have been observed among the negative control group.

### Seropositivity is related to positive T-cell reactivity

In this study, antibody status did seem to be a good predictor of T-cell reactivity, since 18 out of the 20 samples with positive serology gave a T-cell response, and only three out of nine patients who demonstrated no T-cell reactivity had antibodies. The converse, however, was not true since out of the 32 patients who responded in the ELISPOT, only 18 were seropositive. It is difficult to draw conclusions about those patients who were seronegative due to the lag phase of several months between infection with the virus and the appearance of antibodies in the blood (Carter *et al* 2000), and the fact that antibody levels eventually diminish. Thus, negative serology does not mean that the individual has never been infected or is unlikely to possess HPV16-specific memory T cells.

We found that the patients who demonstrated T-cell responses of multiple specificities (patients 6, 11, 12, and 13) were seronegative, whereas those showing far fewer T-cell reactivities had the highest antibody titres (patients 5, 8, 18, 18 and 31). One explanation for these results is that the antibody response is slower than the T-cell response. Perhaps, the women showing multiple T-cell reactivities have only recently encountered the virus, and have large numbers of effector T cells, but the antibody response is not yet established. Conversely, in the other group of women with low T-cell reactivity and high antibody titres, the primary effector T-cell response may have subsided, but the serological response is still present and helping to prevent re-infection. It is difficult to define precisely the relationship between T-cell reactivity and antibody status; more knowledge is required about the relative timescales and roles of the T- and B-cell responses to HPV during natural infection.

In summary, we have shown that ELISPOT assays of IFN-*γ* release are capable of revealing T-cell reactivities to HPV16 antigens in women with cervical dysplasia and can be used to establish the spectrum of T-cell responses induced during natural infection. The ELISPOT assay is becoming established as a good method for charting HPV-specific immunity, both during natural infection and following vaccination (van der Burg *et al*, 2001; de Jong *et al*, 2002, 2004; Steele *et al*, 2002; Baldwin *et al*, 2003; Welters *et al*, 2003; Smith *et al*, 2004; Smyth *et al*, 2004). Setting the vaccination studies aside, there are differences between the conclusions drawn from the studies looking at natural immunity and those obtained in this study. There are several possible explanations for this. Firstly, we have examined responses directly *ex vivo*, which is more likely to be representative of the *in vivo* situation. The majority of the other studies employed an *in vitro* stimulation step prior to ELISPOT analysis. Secondly, we established negative cutoff values using a valid negative control group that obviously minimises the problems associated with nonspecificity. Thirdly, many of the published studies have concentrated on HPV-specific responses in normal individuals. By looking at the healthy population, these studies are likely to be detecting predominantly memory T-cell reactivity, whereas we were studying women with recent or current disease, and are therefore likely to be detecting effector populations as well as memory.

The results from the current study suggest that there are differences in how viral antigens are handled by the immune system during progressive disease of the cervix. In order to define this more precisely, more noninterventional prospective studies will be required, although there are obvious ethical problems associated with this. The vast majority of women in this cohort underwent surgical excision that is likely to have an effect on HPV16 immunity, and so a prospective study would not be possible. Defining the role played by HPV16-specific T cells in the natural history of cervical disease will aid the development of immunological assays to determine the risk of CIN progression, and therefore the management of premalignant and malignant HPV-associated neoplasia.

## Figures and Tables

**Table 1:** Clinical details

**Disease grade**											
**Virgins (*n*=9)**		**No CIN (*n*=12)**		**Low grade (*n*=8)**			**High grade (*n*=16)**			**Cancer (*n*=5)**	
**Patient**	**Age (years)**	**Patient**	**Age (years)**	**Patient**	**Age (years)**	**Histology grade**	**Patient**	**Age (years)**	**Histology grade**	**Patient**	**Age (years)**
1	27	2	30	7	39	CIN I	8	26	CIN III	1	44
2	63	4	37	10	75	CIN I	9	37	CIN II	3	45
3	29	6	44	11	25	CIN I	12	26	CIN II	5	40
4	76	13	52	14	37	CIN I	19	29	CIN III	22	37
5	67	15	37	17	23	CIN I	23	27	CIN III	25	NA
6	42	16	42	21	35	CIN I	26	36	CIN III		
7	35	18	29	24	56	CIN I	29	29	CIN III		
8	78	20	49	28	59	CIN I	31	24	CIN II		
9	21	27	64				32	24	CIN III		
		30	35				33	26	CIN II		
		36	23				34	NA	CIN II		
		39	49				35	47	CIN III		
							37	24	CIN III		
							38	30	CIN III		
							40	32	CIN II		
							41	35	CIN III		
Mean age 45.4 years		Mean age 40.9 years		Mean age 43.6 years			Mean age 30.1 years			Mean age 41.5 years	

Details of the virgins (1–9) and patients (1–41) involved in the study, including their age at the time of sampling, and, for the patients, the grade of cervical neoplasia reported following histological analysis of cervical tissue obtained at the same time.

NA, not available.

**Table 2:** Sequences of the 30–35mer peptides (overlapping by 14–16 amino acids) covering HPV16 E6, E7 and E4 proteins used to screen for CD4+ and CD8+ T-cell responses in ELISPOT assays of IFN-*γ* release

	**aa**
*HPV16 E6 sequences*	
MHQKRTAMFQDPQERPRKLPQLCTELQTTIHDI	1–33
KLPQLCTELQTTIHDIILECVYCKQQLLRREV	18–49
ECVYCKQQLLRREVYDFAFRDLCIVYRDGNPYA	36–68
FRDLCIVYRDGNPYAVCDKCLKFYSKISEYRHY	54–86
KCLKFYSKISEYRHYCYSVYGTTLEQQYNKPLC	72–104
YGTTLEQQYNKPLCDLLIRCINCQKPLCPEEK	91–122
RCINCQKPLCPEEKQRHLDKKQRFHNIRGRWT	109–140
DKKQRFHNIRGRWTGRCMSCCRSSRTRRETQL	127–158

*HPV16 E7 sequences*	
MHGDTPTLHEYMLDLQPETTDLYCYEQLNDSS	1–33
TDLYCYEQLNDSSEEEDEIDGPAGQAEPDRAHYNIV	20–55
AGQAEPDRAHYNIVTFCCKCDSTLRLCVQSTHVDI	42–76
STLRLCVQSTHVDIRTLE DLLMGTLGIVCPICSQKP	63–98

*HPV16 E4 sequences*	
MADPAAATKYPLLKLLGSTWPTTPPRPIPKPSP	1–33
WPTTPPRPIPKPSPWAPKKHRRLSSDQDQSQTPET	20–54
RLSSDQDQSQTPETPATPLSCCTETQWTVLQSSLHL	41–76
TETQWTVLQSSLHLTAHTKDGLTVIVTLHP	63–92
